# Predictive model of sarcopenia in chronic kidney disease: an integrated approach of bioinformatics, machine learning, and clinical validation

**DOI:** 10.3389/fphys.2026.1826313

**Published:** 2026-06-19

**Authors:** Liling Zhang, Tingfan Wang, Di Fan, Santao Ou, Yong Xu

**Affiliations:** 1 Department of Endocrinology and Metabolism, The Affiliated Hospital of Southwest Medical University, Luzhou, Sichuan, China; 2 Sichuan Clinical Research Center for Diabetes and Metabolic Diseases, Luzhou, Sichuan, China; 3 Sichuan Clinical Research Center for Nephropathy, Luzhou, Sichuan, China; 4 Department of Nephrology, The Affiliated Hospital, Southwest Medical University, Luzhou, China; 5 Department of Pediatric Surgery, The Affiliated Hospital, Southwest Medical University, Luzhou, China; 6 Department of Anesthesiology, The Affiliated Hospital, Southwest Medical University, Luzhou, China

**Keywords:** chronic kidney disease, machine learning, prediction, sarcopenia, Shapley Additive Explanations

## Abstract

**Background:**

Sarcopenia represents a prevalent and clinically significant complication of chronic kidney disease (CKD), featuring insidious onset and diagnostic complexity. Elucidating the underlying pathogenic factors of CKD-related sarcopenia and constructing reliable predictive models are critical to optimizing clinical prognosis and patient outcomes.

**Methods:**

This study enrolled two independent cohorts to develop and validate a predictive model for sarcopenia in patients with chronic kidney disease (CKD). The primary cohort comprised 2,979 participants from the National Health and Nutrition Examination Survey (NHANES) database (2011–2018), while the independent external validation cohort consisted of 428 CKD patients recruited from the Affiliated Hospital of Southwest Medical University. Predictive variables were selected via least absolute shrinkage and selection operator (Lasso) regression, followed by multivariable logistic regression to identify independent predictors. The NHANES dataset was randomly partitioned into a training set (n = 2,085) and testing set (n = 894) at a 7:3 ratio. Baseline characteristics were compared across the training, testing, and external validation cohorts to evaluate inter-cohort heterogeneity. Furthermore, five machine learning algorithms, including Gradient Boosting (XGBoost), Logistic Regression (LR), Light Gradient Boosting Machine (LightGBM), Random Forest (RF), and Multilayer Perceptron (MLP), were constructed and hyperparameter-optimized using the training dataset. The best-performing model was selected based on predefined performance metrics and further assessed via ten-fold cross-validation to confirm internal robustness. The optimized model was subsequently validated in the external cohort to evaluate its generalizability to real-world CKD populations. Finally, Shapley Additive Explanations (SHAP) were applied to interpret the model at both the global and individual levels, elucidating the contribution of each predictor to sarcopenia risk.

**Results:**

The final model included 12 predictors, with XGBoost demonstrating robust superiority across cohorts. It achieved an AUC of 0.890 (95% CI: 0.860–0.909) for internal testing set, 0.818 (95% CI: 0.790–0.885) for external validation, and 0.888 (95% CI: 0.880–0.896) in the internal validation. The external Brier score was 0.156, indicating good calibration and reliable risk prediction. Decision curve analysis (DCA) confirmed superior net clinical benefit over a wide threshold range, supporting clinical applicability. SHAP analysis further identified gender, BMI, and weight as the top three dominant predictors.

**Conclusion:**

In this study, we developed an XGBoost-based model to predict sarcopenia in patients with chronic kidney disease (CKD). Importantly, to our knowledge, this is the first study to apply SHAP values to interpret model predictions in the context of CKD-related sarcopenia, thereby significantly enhancing the transparency and interpretability of the predictive model. Critically, independent external validation further confirmed the model’s reliability and robustness, offering a practical tool for the early detection and prevention of sarcopenia and supporting the development of personalized treatment strategies for patients with CKD.

## 1 Introduction

Chronic kidney disease (CKD) is a highly prevalent health condition worldwide, affecting approximately 26 million Americans and more than 800 million people globally. It is characterized by progressive renal dysfunction, often accompanied by complications such as anemia and bone disorders (Murphy et al., 2016; Webster et al., 2017). In recent years, CKD-related sarcopenia has garnered increasing attention due to its detrimental effects on muscle strength and functionality (Wang et al., 2022). CKD-related sarcopenia is characterized by reduced muscle mass, weakened muscle strength, and impaired physical performance (Cruz-Jentoft et al., 2019). Furthermore, its development is multifactorial and influenced by a combination of malnutrition, chronic inflammation, and metabolic abnormalities (Watanabe et al., 2019; Mori, 2021; Bakinowska et al., 2024; Heitman et al., 2024). The interaction of these contributing factors further exacerbates the condition and complicates its treatment.

A study indicated that the prevalence of sarcopenia among CKD patients varies widely, ranging between 5–62.5%, and is influenced by factors such as sample size, CKD stage, and patient age (Barreto Silva et al., 2022). Another study suggested that most sarcopenia patients have difficulties performing daily tasks and have a higher risk of experiencing increased anxiety and depression (Sun et al., 2019). Moreover, the combined effects of sarcopenia and mild kidney dysfunction significantly increase both all-cause and cause-specific mortality rates. Therefore, early detection and prompt management of sarcopenia or kidney dysfunction are essential for reducing mortality risks and increasing the quality of life in older adults (Wu et al., 2024).

The diagnosis of sarcopenia relies primarily on comprehensive assessment tools and physical examinations, including the Strength, Assistance with walking, Rising from a chair, Climbing stairs, and Falls (SARC-F) questionnaire, dual-energy X-ray absorptiometry (DXA), bioelectrical impedance analysis (BIA), magnetic resonance imaging (MRI), and computed tomography (CT) (Cruz-Jentoft et al., 2019). However, these methods are often complex, time-consuming, and inconvenient for routine use. Previous literature suggests that certain laboratory markers, including serum myostatin, adipokines, and myokines levels, the serum cystatin C-to-creatinine ratio, and 25-Hydroxyvitamin D levels may assist in the early identification of sarcopenia (An et al., 2022; Yasar et al., 2022; Hori et al., 2024; Wu et al., 2024). Nevertheless, most of the previous studies were based on single indicators that fail to assess the multifaceted pathological mechanisms of sarcopenia and are influenced by factors such as age, sex, CKD stage, and basal metabolic status, thereby limiting their clinical applicability. Current management of CKD-related sarcopenia primarily includes nutritional support, exercise interventions, and pharmacological therapies (Hoshino, 2021; Noor et al., 2021; Massini et al., 2023; Martino et al., 2024), yet the overall efficacy of these treatments remains suboptimal. Therefore, it is important to establish clinical models to reliably predict CKD-related sarcopenia and introduce tailored interventions at an early stage for its better prevention and treatment.

Machine learning (ML) is an emerging field that has become an indispensable component of modern medical research. As a data-driven subset of artificial intelligence, it employs sophisticated algorithms to autonomously identify hidden patterns within complex datasets. ML has been widely adopted across diverse medical domains, including disease prediction, personalized therapy, and risk stratification. Compared with traditional statistical methods, ML overcomes limitations in integrating large-scale and heterogeneous data, enables dynamic risk prediction, and efficiently identifies high-risk subgroups, making it particularly adept at analyzing complex, multi-dimensional datasets (Shehab et al., 2022; Haug and Drazen, 2023).

This study aimed to establish a clinical prediction model for the early identification of risk factors associated with chronic kidney disease (CKD)-related sarcopenia. While a limited number of prior studies have constructed predictive models for CKD-related sarcopenia, ours systematically develops and screens the optimal model using multiple machine learning algorithms. Furthermore, we conducted independent external validation with external datasets to rigorously confirm the model’s reliability and robustness. Concurrently, we integrated Shapley Additive Explanations (SHAP) to comprehensively elucidate the driving factors behind model outputs and significantly enhancing its clinical interpretability and transparency.

## 2 Materials and methods

### 2.1 Study population

**Figure 1:**
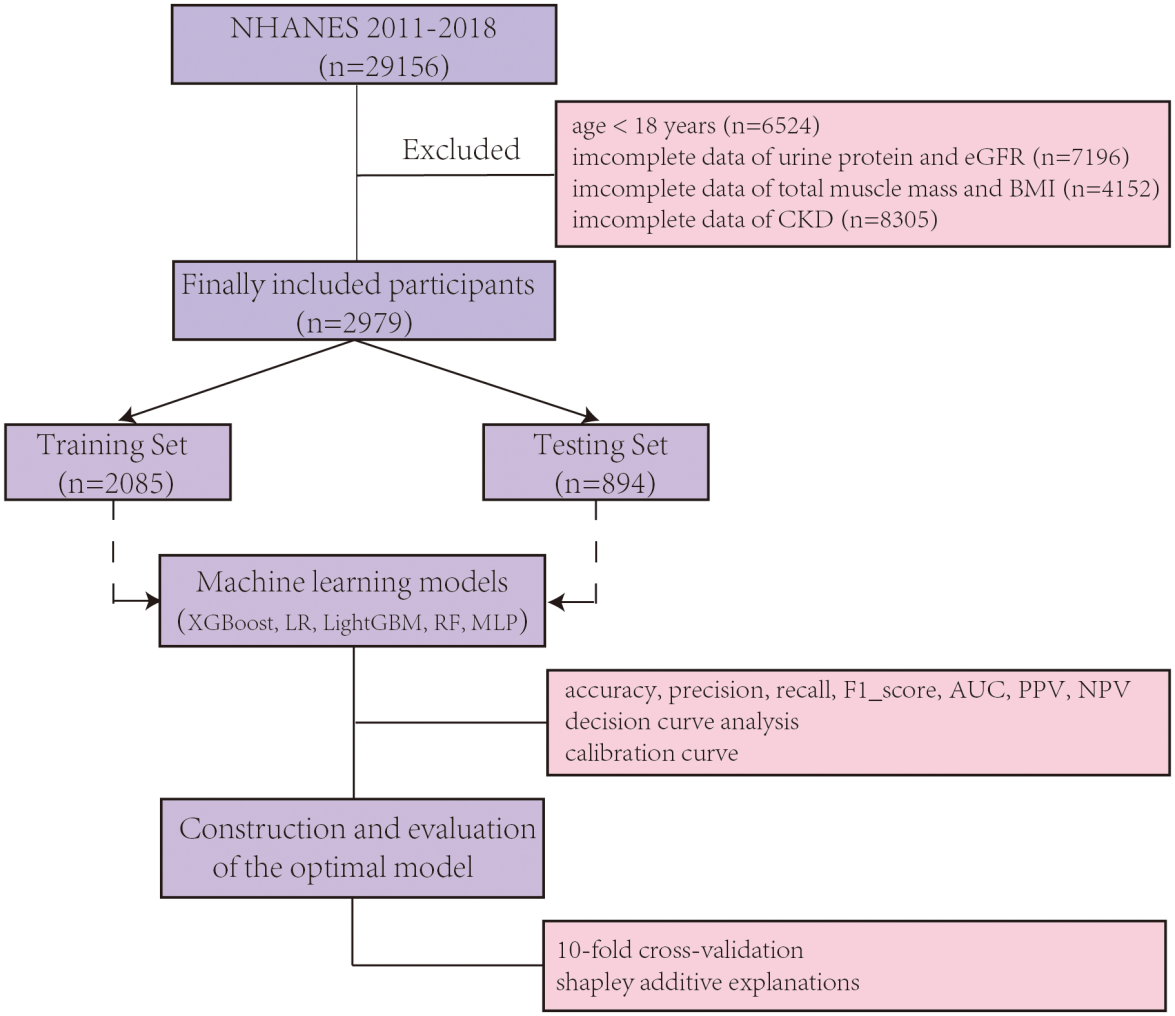
Participant selection and study design flowchart. eGFR, estimated glomerular filtration rate; BMI, body mass index; CKD, chronic kidney disease; LR, logistic regression; RF, random forest; MLP, multilayer perceptron; XGBoost, Gradient Boosting; LightGBM, Light Gradient Boosting Machine.

As illustrated in **Figure 1**, two independent cohorts were enrolled for model development and external validation. A total of 29,156 individuals were initially screened. We excluded participants aged < 18 years (n=6,524), those with missing data on urinary protein and estimated glomerular filtration rate (eGFR) (n=7,196), or lacking total muscle mass and body mass index (BMI) (n=4,152), as well as those not meeting CKD criteria (n=8,305). Finally, 2,979 participants were included in this study. For external validation, an independent set of 428 CKD patients was recruited from the Affiliated Hospital of Southwest Medical University. This external cohort included 121 CKD patients with sarcopenia and 307 non-sarcopenic CKD patients. All participants in the external validation cohort met the standardized diagnostic criteria for CKD and sarcopenia. Diagnosis of CKD: urinary albumin-to-creatinine ratio (UACR) ≥ 30 mg/g and/or eGFR < 60 mL/min/1.73 m². Sarcopenia was defined using an appendicular lean mass (ALM)-to-BMI ratio < 0.789 in males and < 0.512 in females.

### 2.2 Outcome variables

This study included only participants who met the diagnostic criteria for CKD. CKD was defined as a UACR ≥ 30 mg/g and/or eGFR < 60 mL/min/1.73 m². The CKD-EPI equation was employed to calculate eGFR: For males with ≤ 0.9 mg/dL serum creatinine: GFR = 141 × (serum creatinine/0.9)^-0.411^ × (0.993)^age^, whereas for males with > 0.9 mg/dL serum creatinine: GFR = 141 × (serum creatinine/0.9)^-1.209^ × (0.993)^age^. For females with ≤ 0.7 mg/dL serum creatinine: GFR = 144 × (serum creatinine/0.7)^-0.329^ × (0.993)^age,^ while for females with > 0.7 mg/dL serum creatinine: GFR = 144 × (serum creatinine/0.7)^-1.209^ × (0.993)^age^. The UACR was calculated using the formula: UACR (mg/g) = urinary albumin (mg/dL)/urinary creatinine (g/dL). Moreover, sarcopenia was assessed using ALM adjusted for BMI. The diagnostic criteria were ALM/BMI < 0.789 for males and ALM/BMI < 0.512 for females.

### 2.3 Predictor variables

This study included 28 potential predictor variables derived from three sources. Demographic and examination variables included gender, age, BMI, weight, waist circumference, systolic blood pressure (SBP), and diastolic blood pressure (DBP). Laboratory variables comprised white blood cell (WBC), lymphocyte (LYM), monocyte (MON), neutrophil (NEU), platelet (PLT), lymphocyte percentage (LYM%), monocyte percentage (MON%), neutrophil percentage (NEU%), eosinophil percentage (EOS%), systemic inflammation response index [SIRI, (NEU × MON)/LYM] and systemic immune-inflammation index [SII, (PLT × NEU)/LYM]. Other laboratory measures included serum creatinine (SCR), total protein (TP), albumin (ALB), globulin (GB), alkaline phosphatase (ALP), creatine phosphokinase (CPK), gamma-glutamyl transferase (γ-GGT), and glycated hemoglobin (HbA1c). The data on diabetes and smoking status (never, former, or current: defined by whether participants had smoked at least 100 cigarettes in their lifetime and if they were currently smoking) were acquired using the Questionnaire.

### 2.4 Construction and evaluation of the prediction model

To eliminate data leakage, we adopted a split-first analytical strategy. The overall cohort derived from NHANES was randomly split into training and internal test sets at a 7:3 ratio, with a separate independent external validation cohort. Baseline characteristics of the three cohorts were compared to ensure comparability. All variable selection, model training, and hyperparameter tuning were strictly confined to the training set; the testing and external validation sets were only used for final evaluation. Candidate predictors were screened via Lasso regression to reduce dimensionality and multicollinearity. Variables with non-zero Lasso coefficients were further assessed by multivariate logistic regression, and 12 independent predictors were finally identified. Five machine learning models were applied: XGBoost, LR, LightGBM, RF and MLP. Class-weighted training was used to handle outcome imbalance. Optimal hyperparameters were determined by Bayesian optimization coupled with 5-fold cross-validation in the training set, and nested cross-validation was considered to improve model reliability. Model performance was evaluated via accuracy, precision, recall, F1-score and AUC with bootstrap 95% CIs. Calibration curves and DCA were used to assess model calibration and clinical utility. Internal ten-fold cross-validation was performed on the training set, followed by external validation to verify generalizability. SHAP analysis was conducted to interpret model outputs, rank feature importance and enable individual-level predictive explanation.

### 2.5 Statistical methods

Continuous variables with a normal distribution were expressed as mean ± standard deviation and compared using independent samples t-tests. Non-normally distributed variables were reported as medians and interquartile ranges and compared using the Mann-Whitney U test. Categorical variables were summarized as counts (percentages) and analyzed via the chi-square test. All statistical analyses were performed using R (version 4.3.3) and Python (version 3.11.8), with a two-tailed P-value < 0.05 indicating statistical significance.

## 3 Results

### 3.1 Baseline participant characteristics

This study included 2,979 CKD-diagnosed participants. During the modeling process, all participants were randomly assigned to a training set (n = 2085; 371 with sarcopenia, 1714 without sarcopenia) and a testing set (n = 894; 159 with sarcopenia, 735 without sarcopenia) in a 7:3 ratio. An independent external validation cohort was also recruited (n = 428; 121 with sarcopenia, 307 without sarcopenia). Baseline characteristics were compared across the training, testing, and external validation sets, with no statistically significant differences observed among the three groups (P > 0.05). Detailed baseline information is summarized in **Table 1**.

**Table 1:** Baseline characteristics of the training set, testing set, and external validation set.

Variable	Training set (n =2085)	Testing set (n = 894)	External validation set (n = 428)	P-value
Age, Median (SD)	39.13 (11.95)	38.94 (11.58)	39.54 (10.32)	0.606
Weight, Median (SD)	76.41 (22.28)	77.89 (22.12)	68.45 (27.87)	0.091
Waist, Median (SD)	96.55 (17.44)	97.50 (17.70)	95.56 (15.86)	0.354
BMI, Median (SD)	29.26 (7.52)	29.24 (7.49)	28.66 (18.77)	0.930
SBP, Median (SD)	119.06 (17.15)	119.98 (16.77)	121.87 (18.99)	0.188
DBP, Median (SD)	71.18 (11.46)	71.54 (11.43)	73.08 (15.76)	0.479
WBC, Median (SD)	7.51 (2.17)	7.47 (2.25)	7.89 (3.76)	0.613
LYM, Median (SD)	2.29 (0.72)	2.27 (0.70)	2.68 (0.81)	0.544
MON, Median (Q1, Q3)	0.50 (0.40, 0.60)	0.50(0.40, 0.60)	0.48 (0.39, 0.73)	0.978
NEN, Median (SD)	4.45 (1.71)	4.42 (1.73)	4.50 (1.87)	0.729
SIRI, Median (Q1, Q3)	0.97 (0.67, 1.40)	0.97 (0.65, 1.40)	0.98 (0.64, 1.54)	0.940
SII, Median (Q1, Q3)	477.98 (345.83, 651.23)	484.09 (336.77, 660.59)	480.87 (350.67, 658.80)	0.606
LYMe %, Median (SD)	31.30 (7.94)	31.32 (7.53)	32.10 (8.43)	0.929
MON %, Median (SD)	7.32 (1.90)	7.36 (1.93)	7.32 (1.99)	0.694
NEU %, Median (SD)	58.22 (8.78)	58.12 (8.27)	59.76 (9.43)	0.776
EOS %, Median (SD)	2.00 (1.30, 3.20)	2.00 (1.30, 3.20)	2.03 (1.31, 3.40)	0.601
PLT, Median (SD)	257.07 (63.94)	257.35 (66.41)	257.89 (65.87)	0.918
SCR, Median (SD)	58.32 (15.90)	58.83 (16.51)	59.33 (17.34)	0.525
ALB, Median (SD)	42.03 (3.32)	42.13 (3.31)	43.19 (3.79)	0.465
ALP, Median (Q1, Q3)	67.00 (54.00, 83.00)	67.00 (54.00, 81.00)	67.54 (53.04, 85.32)	0.588
CPK, Median (Q1, Q3)	90.00 (65.00, 127.00)	92.00 (66.00, 129.00)	91.00 (59.00, 131.00)	0.296
γ-GGT, Median (Q1, Q3)	18.00 (12.25, 29.00)	18.00 (13.00, 27.00)	18.90 (12.90, 28.54)	0.691
TP, Median (SD)	72.12 (4.49)	72.21 (4.50)	72.11 (4.50)	0.523
GB, Median (SD)	30.09 (4.41)	30.07 (4.26)	30.38 (5.09)	0.900
HbA1c, Median (Q1, Q3)	5.40 (5.20, 5.80)	5.40 (5.20, 5.80)	5.39 (5.18, 5.98)	0.807
Gender, n(%)				0.753
Male	1721 (82.55%)	734 (82.08%)	303 (70.8%)	
Female	364 (17.45%)	160 (17.92%)	125 (29.2%)	
Smoking, n(%)				0.157
Currently smoking	674 (32.36%)	261 (29.12%)	106 (24.77%)	
Never smoking	1409 (67.55%)	633 (70.88%)	319 (74.53%)	
Former smoking	2 (0.10%)	0 (0.00%)	3 (0.7%)	
Diabetes, n(%)				0.844
No	1842 (88.35%)	788 (88.13%)	366 (85.51%)	
Yes	243 (11.65%)	106 (11.87%)	62 (14.49%)	

Continuous variables are expressed as the median. Categorical variables are expressed as percentages; significant at p < 0.05. BMI, body mass index; SBP, systolic blood pressure; DBP, diastolic blood pressure; WBC, white blood cell count; LYM, lymphocyte count; MON, monocyte count; NEU, neutrophil count; PLT, platelet; LYM%, lymphocyte percentage; MON%, monocyte percentage; NEU%, neutrophil percentage; EOS%, eosinophil percentage; SIRI, systemic inflammation response index; SII, systemic immune-inflammation index; SCR, serum creatinine; TP, total protein; ALB, albumin; GB, globulin; ALP, alkaline phosphatase; CPK, creatine phosphokinase; γ–GGT, gamma-glutamyl transferase; HbA1c, glycated hemoglobin.

### 3.2 Selection of feature variables

**Figure 2:**
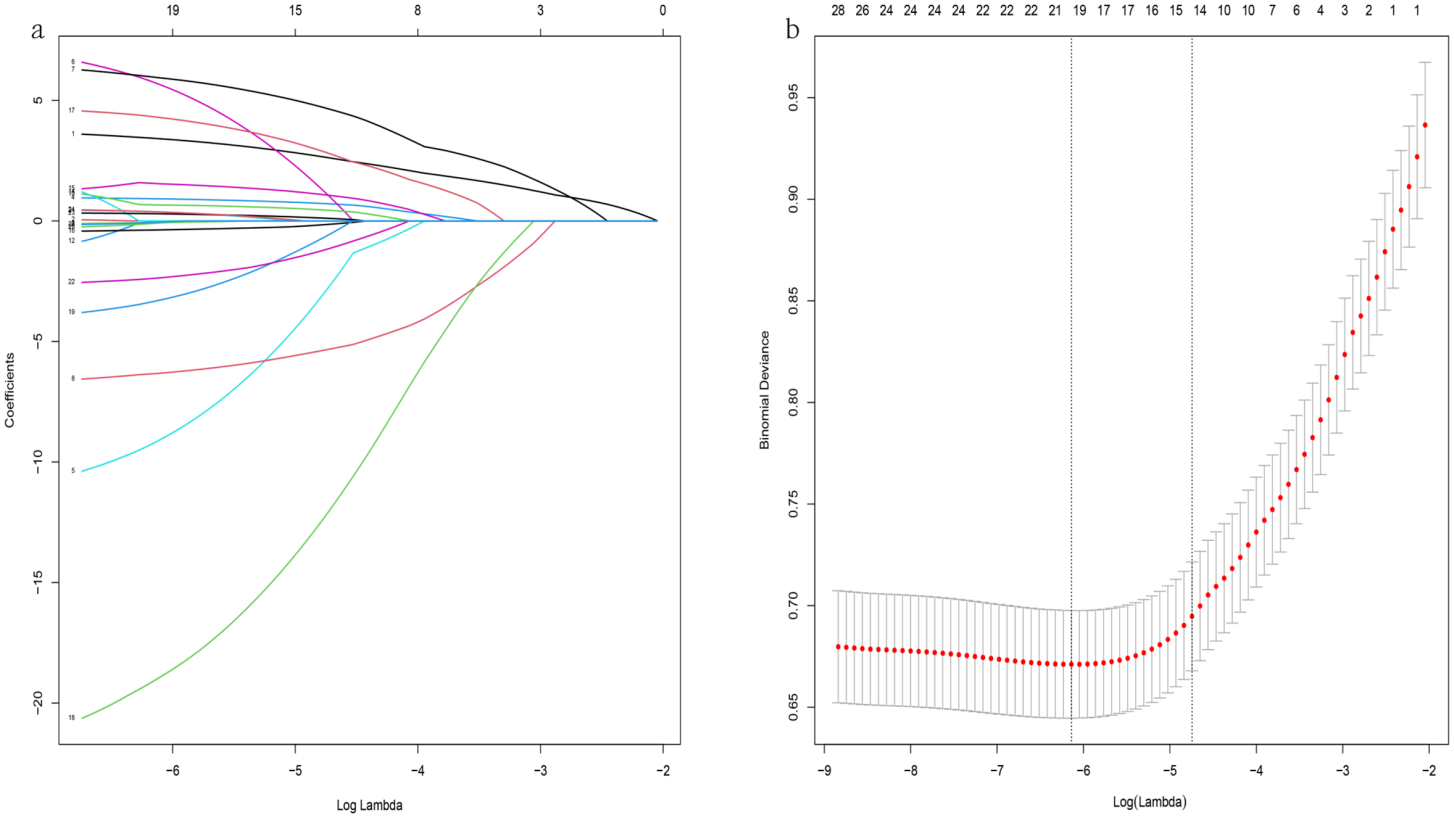
Lasso regression for feature selection. **(a)** Coefficient profile plot of Lasso regression. **(b)** 20-fold cross-validation curve for Lasso regression. Dotted vertical lines indicate the optimal λ values (λ.min = 0.0022 and λ.1SE = 0.0087).

Sarcopenia was defined as the dependent variable, with all other variables considered independent for Lasso regression analysis. Lasso regression was used for variable screening to reduce dimensionality and mitigate multicollinearity. The optimal tuning parameter was determined via 20-fold cross-validation, which identified 14 features with non-zero coefficients (**Figure 2**): gender, age, weight, waist, BMI, SBP, HbA1c, ALB, PLT, CPK, ALP, γ-GGT, SCR, and LYM. These 14 variables were further incorporated into multivariable logistic regression to identify independent predictive factors. Ultimately, 12 variables including gender, age, weight, waist circumference, BMI, SBP, HbA1c, PLT, CPK, ALP, γ-GGT, and SCR were selected as the final predictors (all P < 0.05), as presented in **Table 2**.

**Table 2:** Multivariate logistic regression analysis.

Variable	R	SE	Z	P-value	OR (95% CI)
(Intercept)	-6.39	1.19	-5.35	<0.001	(-)
Gender	3.78	0.19	20.15	<0.001	43.97 (30.43-63.54)
Age	0.03	0.01	4.73	<0.001	1.03 (1.02-1.04)
Weight	-0.08	0.01	-10.15	<0.001	0.92 (0.91-0.94)
Waist	0.08	0.01	8.46	<0.001	1.09 (1.07-1.11)
BMI	0.12	0.01	11.06	<0.001	1.13 (1.10-1.15)
SCR	-0.04	0.01	-8.01	<0.001	0.96 (0.95-0.97)
LYM	0.09	0.09	1.10	0.271	1.10 (0.93-1.30)
PLT	0.003	0.001	3.03	0.002	1.00 (1.00-1.00)
ALB	-0.02	0.02	-0.87	0.384	0.98 (0.94-1.02)
ALP	0.01	0.003	4.26	<0.001	1.01 (1.01-1.02)
CPK	-0.008	0.001	-7.25	<0.001	0.99 (0.99-0.99)
γ-GGT	-0.003	0.001	-2.63	0.009	1.00 (0.99-1.00)
HbA1c	-0.20	0.04	-4.57	<0.001	0.82 (0.75-0.89)
SBP	0.007	0.004	2.05	0.040	1.01 (1.00-1.01)

significant at P < 0.05. BMI, body mass index; LYM, lymphocyte count; PLT, platelet; SCR, serum creatinine; ALB, albumin; ALP, alkaline phosphatase; CPK, creatine phosphokinase; γ-GGT, gamma-glutamyl transferase; HbA1c, glycated hemoglobin; SBP, systolic blood pressure; R, regression coefficient; SE, standard error; OR, odds ratio; CI, confidence interval.

### 3.3 Model development and evaluation

**Figure 3:**
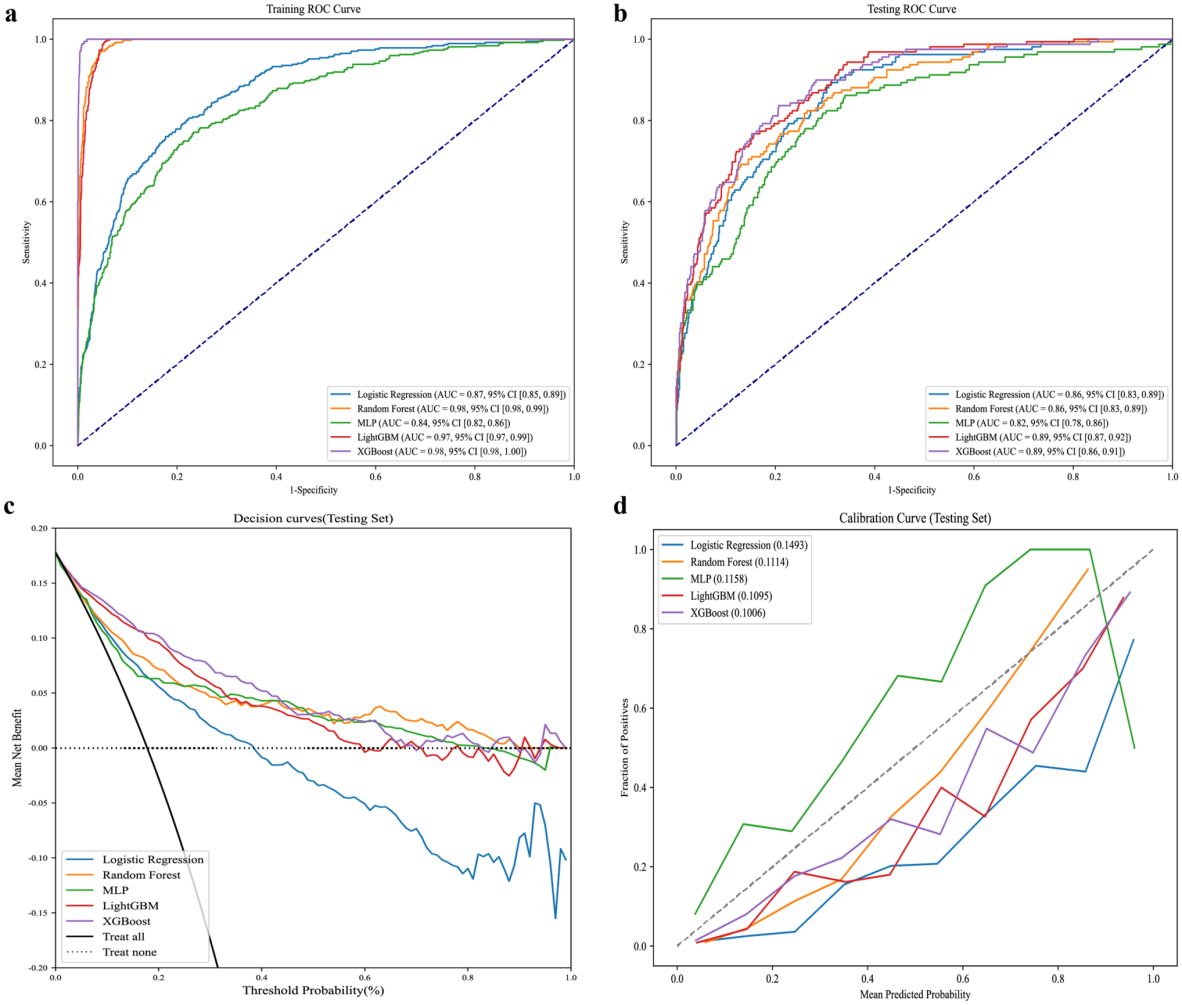
The ROC of models in training set and testing set, decision curve analysis and calibration curve in testing set. **(a)** ROC in training set. **(b)** ROC in testing set. **(c)** DCA in testing set. **(d)** calibration curves in testing set. Abbreviations: LR, logistic regression; RF, random forest; MLP, multilayer perceptron; XGBoost, Gradient Boosting; LightGBM, Light Gradient Boosting Machine.

XGBoost achieved the highest discriminative performance on the training set (AUC: 0.98, 95% CI: 0.98–1.00), though with a substantial drop in the testing set, yielding an AUC of 0.89 (95% CI: 0.86–0.91) and a recall of 0.65 (95% CI: 0.57–0.73). Despite this decline, it remained the best-performing model in the testing cohort. Furthermore, MLP showed the weakest generalization, with an AUC of 0.82 (95% CI: 0.78–0.86) and a recall of 0.32 (95% CI: 0.24–0.41) (**Table 3** and **Figures 3a,b**). Given that screening models should prioritize sensitivity, we supplemented AUC with threshold trade-off analysis and confusion matrices. We report both the threshold maximizing the Youden index and a sensitivity–prioritized threshold suitable for screening scenarios (Supporting files). Notably, DCA revealed that treatment strategies derived from all five machine learning models were superior to default approaches (**Figure 3c**). Finally, calibration curve analysis confirmed that XGBoost exhibited the best calibration among all models, with the smallest deviation between observed and ideal curves (Brier score: 0.10) (**Figure 3**).

**Table 3:** Comparison of the performance of five models.

Methods	Precision	Recall	Specificity	F1_score	AUC	PR-AUC	Brier-Score	PPV	NPV	MCC
Training set										
LR	0.46(0.42-0.50)	0.78(0.73-0.82)	0.80(0.78-0.82)	0.58(0.54-0.61)	0.87(0.85-0.89)	0.63(0.58-0.68)	0.14(0.13-0.15)	0.46(0.42-0.50)	0.94(0.93-0.95)	0.48(0.44-0.52)
RF	0.85(0.81-0.88)	0.94(0.92-0.97)	0.96(0.95-0.97)	0.89(0.87-0.91)	0.98(0.98-0.99)	0.96(0.94-0.98)	0.60(0.05-0.06)	0.85(0.81-0.88)	0.99(0.98-0.99)	0.87(0.84-0.89)
MLP	0.82(0.74-0.90)	0.21(0.16-0.25)	0.99(0.98-0.99)	0.33(0.27-0.39)	0.84(0.82-0.86)	0.59(0.54-0.64)	0.11(0.10-0.12)	0.82(0.74-0.90)	0.85(0.84-0.87)	0.36(0.30-0.42)
LightGBM	0.73(0.70-0.77)	0.99(0.98-0.99)	0.92(0.91-0.93)	0.85(0.82-0.87)	0.97(0.97-0.99)	0.95(0.94-0.97)	0.06(0.05-0.06)	0.99(0.98-1.00)	0.73(0.70-0.77)	0.99(0.99-0.99)
XGBoost	0.85(0.82-0.89)	0.99(0.99-1.00)	0.96(0.95-0.97)	0.92(0.90-0.94)	0.98(0.98-1.00)	0.97(0.96-0.98)	0.03(0.03-0.03)	0.98(0.96-0.99)	1.00(1.00-1.00)	0.90(0.88-0.93)
Testing set										
LR	0.45(0.39-0.51)	0.72(0.64-0.79)	0.81(0.78-0.83)	0.55(0.49-0.61)	0.86(0.83-0.89)	0.70(0.63-0.78)	0.15(0.14-0.16)	0.45(0.39-0.51)	0.93(0.91-0.95)	0.44(0.38-0.51)
RF	0.61(0.53-0.70)	0.51(0.43-0.59)	0.93(0.91-0.95)	0.56(0.48-0.63)	0.86(0.83-0.89)	0.74(0.66-0.81)	0.11(0.10-0.12)	0.61(0.53-0.70)	0.90(0.88-0.92)	0.47(0.39-0.55)
MLP	0.84(0.70-0.95)	0.20(0.14-0.27)	0.99(0.98-1.00)	0.32(0.24-0.41)	0.82(0.78-0.86)	0.67(0.60-0.75)	0.12(0.10-0.13)	0.84(0.70-0.95)	0.85(0.83-0.87)	0.37(0.28-0.44)
LightGBM	0.55(0.49-0.62)	0.72(0.65-0.79)	0.87(0.85-0.90)	0.63(0.57-0.68)	0.89(0.87-0.92)	0.78(0.71-0.85)	0.11(0.10-0.12)	0.55(0.49-0.62)	0.94(0.92-0.95)	0.54(0.47-0.60)
XGBoost	0.58(0.51-0.65)	0.65(0.57-0.73)	0.90(0.88-0.92)	0.61(0.55-0.67)	0.89(0.86-0.91)	0.79(0.73-0.86)	0.10(0.09-0.11)	0.58(0.50-0.65)	0.92(0.90-0.94)	0.52(0.45-0.59)

LR, logistic regression; RF, random forest; MLP, multilayer perceptron; XGBoost, Gradient Boosting; LightGBM, Light Gradient Boosting Machine; AUC: area under the curve; PR-AUC: Precision-Recall Area Under the Curve; PPV: Positive predictive value; NPV: negative predictive value; MCC: Matthews Correlation Coefficient.

### 3.4 Construction and evaluation of the optimal model

**Figure 4:**
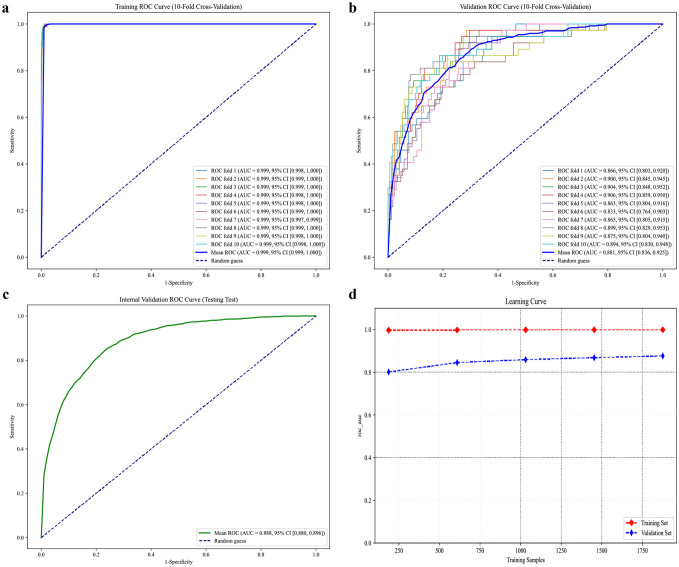
The ROC and learning curve of XGBoost model in training set, validation set, and testing set. **(a)** ROC curves for each fold of 10-fold cross-validation in the training subset, with fold-specific AUC and 95% CI values. **(b)** Corresponding validation ROC curves for each fold of 10-fold cross-validation, with fold-specific AUC and 95% CI values. **(c)** ROC curve for model evaluation in the independent testing set, with the overall AUC and 95% CI. **(d)** Learning curve. The red dashed line represents the training set and the blue dashed line represents the validation set (where 10% of the training set in each round of cross-validation is partitioned as the validation set).

XGBoost was employed to build a predictive model using the NHANES-derived primary training set (n=2,085), with 10-fold cross-validation (CV) implemented to comprehensively evaluate its performance. During the 10-fold CV process, the model achieved a mean AUC of 0.999 (95% CI: 0.999–1.000) for the training subsets and 0.881 (95% CI: 0.836–0.925) for the validation subsets, respectively. Furthermore, the finalized model was tested on an independent internal testing set (n=894), it yielded a final AUC of 0.888 (95% CI: 0.880–0.896) (**Figures 4a–c**). Notably, the AUC of the CV validation subsets was only marginally lower than that of the independent internal testing set, with a relative difference of less than 5%. This minimal relative difference indicates that the XGBoost model possesses favorable generalization ability. Additionally, both the training and validation subsets demonstrated a high degree of goodness-of-fit and strong stability (**Figure 4d**). Collectively, these findings demonstrate that the XGBoost model exhibits excellent reliability and practical applicability for the predictive objectives of this study.

### 3.5 External validation of the optimal model

**Figure 5:**
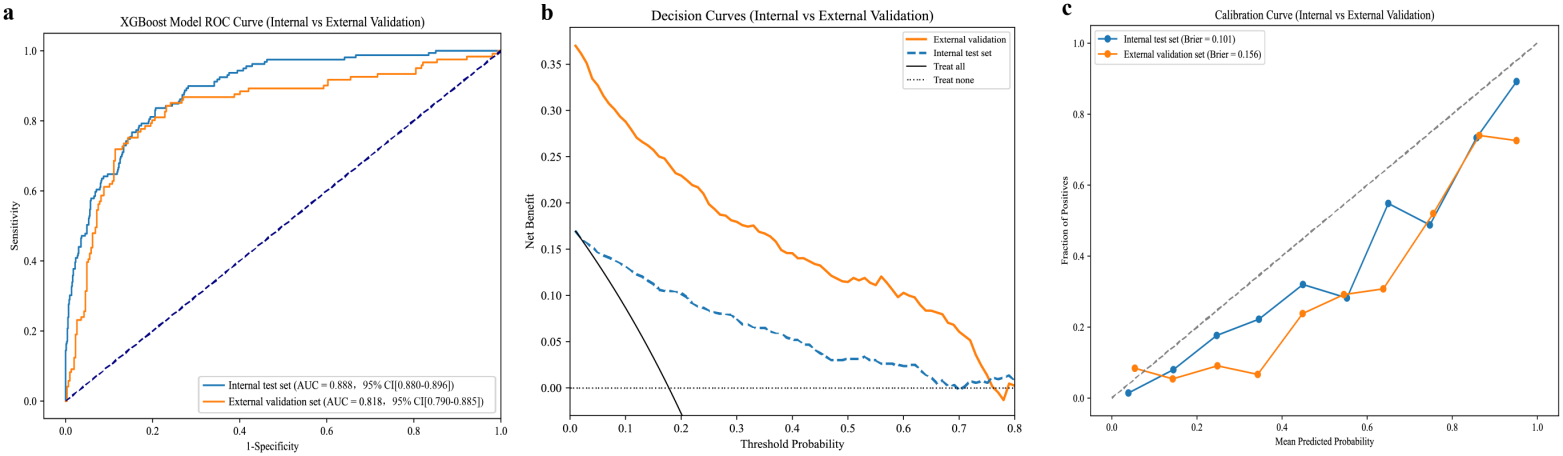
Performance of the XGBoost model in internal and external validation. **(a)** ROC curves of the model in internal and external validation with corresponding AUC and 95% CI value. **(b)** DCA showing the net benefit of the model across different threshold probabilities in external validation and internal test. **(c)** Calibration curves of the model in external validation and internal test, with the 45° dashed line representing perfect calibration. The Brier score is reported for each validation set to assess predictive accuracy.

To further evaluate the generalizability and clinical utility of the optimized XGBoost model, independent external validation was performed in a cohort of 428 patients from the Affiliated Hospital of Southwest Medical University. As illustrated in the **Figure 5a**, the model achieved an AUC of 0.818 (95% CI: 0.790–0.885) in this external cohort, maintaining satisfactory discriminative performance and aligning favorably with the internal testing set (AUC = 0.888, 95% CI: 0.880–0.896). DCA confirmed consistent positive net benefit across all threshold probabilities in the external cohort (**Figure 5b**). For calibration, the Brier score was 0.156; a modest increase relative to internal data indicated slight attenuation of calibration in an independent population, yet remained clinically acceptable (**Figure 5c**). The Brier score ranges from 0 to 1, with lower values indicating better predictive accuracy; a score < 0.2 is generally considered favorable for clinical prediction models. Collectively, these results demonstrate that the XGBoost model retains stable discrimination, acceptable calibration, and positive clinical net benefit in an independent external CKD cohort, supporting its robust generalizability and potential clinical value for sarcopenia prediction in patients with CKD.

### 3.6 SHAP analysis results and feature importance visualization

**Figure 6:**
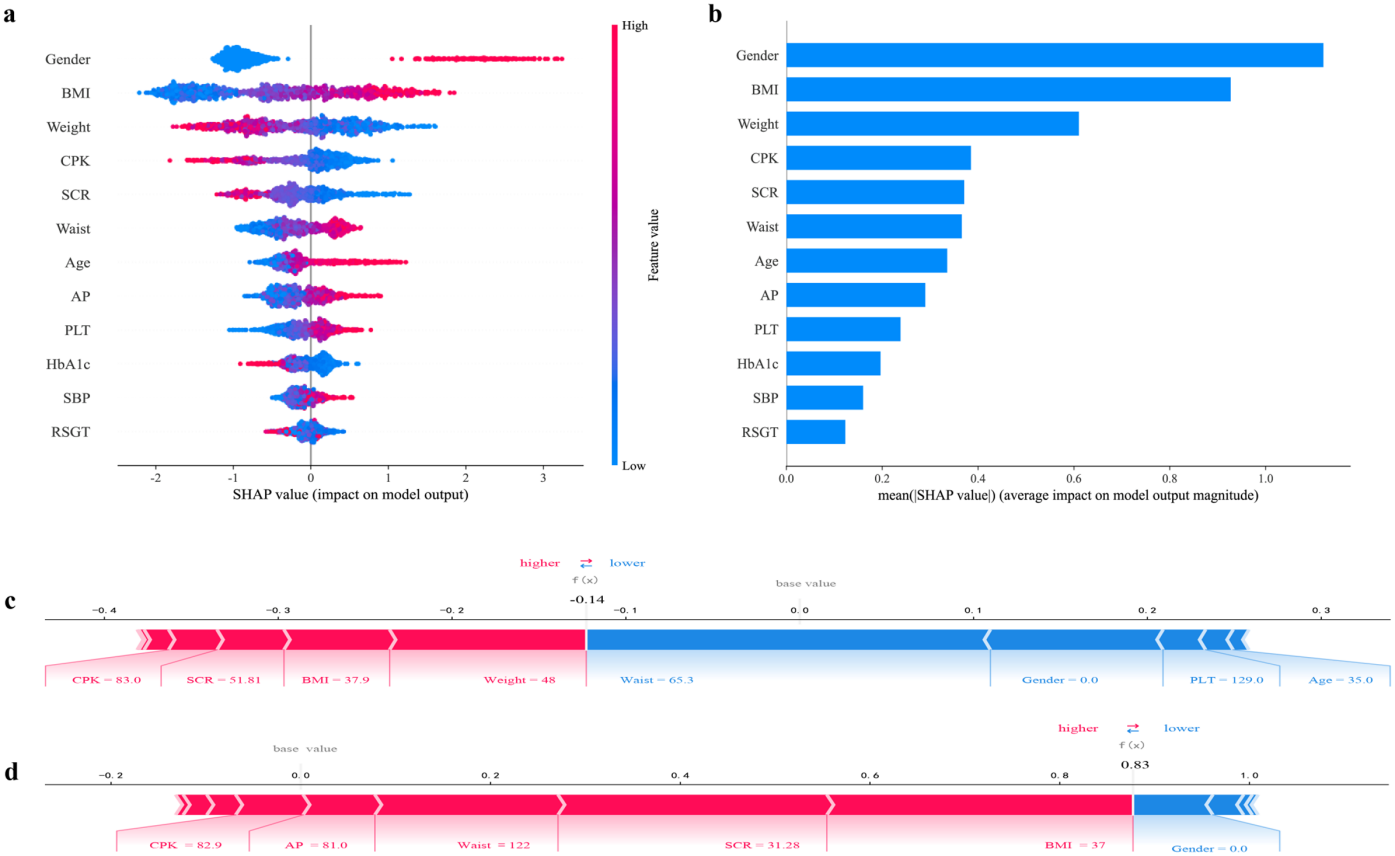
SHAP interprets the model. **(a)** The SHAP values were employed to explain the positive and negative impacts of features for the prediction of sarcopenia. **(b)** The weights of variables importance. **(c)** Individual efforts by patients without sarcopenia. **(d)** Individual efforts by patients with sarcopenia. F(x) represents the logarithmic ratio of each observation, the red features denote the characteristics that increase the risk of sarcopenia, while the blue features represent those that reduce the risk of sarcopenia.

The SHAP analysis identified gender as the strongest predictor of sarcopenia outcomes, followed by BMI, weight, CPK, SCR, and waist circumference. Furthermore, age, SBP, HbA1c, PLT, ALP, and γ-GGT were identified to have relatively lower predictive values (**Figure 6a**). The SHAP summary plot indicates the influence and direction of each feature on model predictions (**Figure 6b**) and reveals that female gender, higher BMI and waist circumference, advanced age, increased platelet count, elevated alkaline phosphatase levels, and higher systolic blood pressure are associated with an increased risk of sarcopenia in patients with CKD. Whereas elevated levels of weight, CPK, SCR, HbA1c, and γ-GGT were linked with a reduced risk of developing sarcopenia. Similarly, individual force plots indicate the contribution of each feature to positive and negative outcome predictions (**Figure 6c**). For patients without sarcopenia, the SHAP prediction score was low (-0.14), whereas it was higher (0.83) for those with sarcopenia (**Figure 6d**).

## 4 Discussion

Sarcopenia was initially defined as an age-related decline in skeletal muscle mass; however, it has been redefined as a musculoskeletal disorder characterized by reduced muscle mass and decreased muscle strength and physical function (Rosenberg, 1997; Cruz-Jentoft and Sayer, 2019). CKD, as a state of accelerated aging, gradually reduces physical function and nutritional abnormalities, thereby significantly increasing the risk of sarcopenia in affected patients. Furthermore, sarcopenia increases CKD progression, the risk of mortality, and advancement to end-stage renal disease (Wilkinson et al., 2021; Ribeiro et al., 2022; Shu et al., 2022; Nakano et al., 2024; Wu et al., 2024). The pathogenesis of CKD-related sarcopenia is multifactorial, including chronic low-grade inflammation, insulin resistance, metabolic disturbances, and heightened oxidative stress, all of which may cause excessive protein degradation and the production of pro-inflammatory factors (Watanabe et al., 2019; Mori, 2021; Andres-Hernando et al., 2023; Zhang et al., 2023; Bakinowska et al., 2024; Heitman et al., 2024; Wu et al., 2024). Moreover, most CKD patients have gut microbiota dysbiosis and abnormal serum metabolites, which further disrupt metabolic homeostasis, exacerbating inflammatory responses and ultimately inducing sarcopenia (Bakinowska et al., 2024; Zheng et al., 2024). Accumulated evidence from systematic reviews has confirmed a high prevalence of sarcopenia among CKD populations, particularly in dialysis patients (Duarte et al., 2024). This study novelty constructed and validated an XGBoost model for CKD-related sarcopenia, with external validation incorporated and model interpretability further evaluated based on SHAP analysis. Specifically, the XGBoost model exhibited good discriminatory performance, with an internal validation AUC of 0.888 and an external validation AUC of 0.818, while acceptable calibration was also achieved with an external Brier score of 0.156.

Machine learning is a powerful tool for optimizing diagnostic processes, enhancing clinical efficiency, and reducing healthcare costs. This study developed an AI-based model to predict CKD-related sarcopenia for the first time using a machine-learning approach. The results demonstrated that the XGBoost model performed the best in predicting CKD-related sarcopenia, indicating promising potential for clinical application. The XGBoost algorithm is based on the optimization of gradient-boosting decision trees, which makes it particularly well-suited for large datasets and complex feature spaces. In recent years, XGBoost-based prediction models have been widely applied in various medical fields, such as sepsis, cardiovascular diseases, and diabetes-related insulin resistance, consistently demonstrating excellent predictive performance (Hou et al., 2020; Li et al., 2022; Tsai et al., 2023). In comparison to the traditional logistic regression algorithms, XGBoost can effectively identify nonlinear relationships and build the final model by integrating multiple weak classifiers, thus significantly improving generalization capabilities. Further, it can automatically handle missing values, eliminating the need for complex data preprocessing. Furthermore, it is also highly robust to outliers and noisy data, which minimizes the impact of data noise on model performance.

Although machine learning models have high predictive accuracy, their lack of intuitive explanations, which clarify how individual variables specifically influence prediction outcomes, limits their widespread clinical application. SHAP combines optimal credit allocation with local interpretability, thereby comprehensively indicating the importance of each variable in the model and providing more interpretable outputs (Lundberg et al., 2020). This study revealed that sex was the most significant predictor of sarcopenia, followed by BMI, body weight, CPK, SCR, and waist circumference. Previous literature has reported no significant difference in the prevalence of CKD-related sarcopenia between men and women (Duarte et al., 2024). However, a recent systematic review analyzed the sex-specific effects and showed that female CKD patients were more susceptible to developing sarcopenia (Troutman et al., 2024). Furthermore, a study from Taiwan highlighted that male and age were independent risk factors for sarcopenia in CKD patients not undergoing dialysis (Huang et al., 2024). These studies suggest that the male and female’s skeletal muscle responses to the CKD pathophysiology may have significant differences. Potential mechanisms include sex-related differences in muscle atrophy, muscle composition, anabolic and catabolic pathways, hormonal interactions, and mitochondrial function (Rosa-Caldwell and Greene, 2019; Zhang et al., 2023). Several studies have assessed the influence of obesity-related indicators such as BMI, body weight, and waist circumference on sarcopenia development and revealed that low BMI, malnutrition, and a heightened inflammatory state are key risk factors for CKD-related sarcopenia (Chatzipetrou et al., 2022). Gungor et al. reported a significant positive association between appendicular skeletal muscle mass and factors including body weight, normally hydrated body weight, height, and BMI in CKD patients, which is consistent with this study. The underlying mechanism may involve insulin resistance, which is closely associated with obesity. In CKD patients, insulin resistance has been found to accelerate muscle catabolism and inhibit muscle anabolism, which potentially explains the paradoxical phenomenon of muscle loss in obese patients despite weight gain, known as “sarcopenic obesity” (Gungor et al., 2021). Overall, this study utilized a machine learning model to identify risk factors significantly associated with CKD-related sarcopenia, and the results were consistent with previous studies that used traditional statistical methods. This study provided further insights into the mechanisms underlying CKD-related sarcopenia and offered valuable references for personalized interventions.

There are several limitations in this study. First, the single-center cross-sectional design of this study limits causal inference. Therefore, further multicenter longitudinal studies are warranted to more comprehensively investigate the risk factors for CKD-related sarcopenia. Furthermore, due to inherent limitations of the database, some key variables such as interleukin-6, tumor necrosis factor-α, prealbumin and transferrin may not have been included in the analysis, which may compromise the comprehensiveness of the model. Nevertheless, the established model offers a valuable tool for clinicians, allowing timely identification of patients with CKD-related sarcopenia and supporting the development and optimization of individualized treatment strategies.

## 5 Conclusion

In this study, we developed and externally validated an XGBoost-based predictive model for sarcopenia in patients with CKD using 12 routinely available clinical indicators. The model demonstrated excellent discriminative ability, satisfactory calibration, and robust clinical utility in both internal testing and an independent external validation cohort. Furthermore, SHAP analysis was conducted to quantify the relative importance and directional effects of each predictor, substantially enhancing model interpretability. This tool enables early detection, risk stratification, and timely intervention for CKD-related sarcopenia. Future multicenter longitudinal studies incorporating additional potential biomarkers are warranted to further refine the model and improve its generalizability and clinical applicability.

## Data Availability

The original contributions presented in the study are included in the article/supplementary material. Further inquiries can be directed to the corresponding authors.
